# The Book World of Medicine and Science

**Published:** 1911-09-02

**Authors:** 


### The Book World of Medicine and Science.

Essentials of Histology. By E. A. Schäfer, M.D., Sc.D., LL.D., F.R.S. (London: Longmans, Green and Co. Pp. 571. Price 10s. 6d. net.)

This well-known work has now reached its eighth edition, and that fact in itself bears ample proof, if proof were necessary, of the popularity that the book has attained. The form adopted in the writing of the work is as in previous editions. It is divided into fifty lessons, and so each section treated is complete in itself. It is rather larger than previous issues, but this is mainly due to the introduction of additional illustrations, many of which are actual photographs of microscopic preparations, and we strongly recommend the volume as one of the best text-books of its kind that we know.

Public Health and National Insurance. By H. Meredith Richards, M.D. (London: P. S. King and Co. 1911. Price 6d. net.)

This reprint of articles contributed both to the medical and lay Press by Dr. Richards, who is Medical Officer of Health and School Medical Officer at Croydon, is an admirable pamphlet to place in the hands of anyone. whether a doctor or not, who wants a calm and dispassionate survey of the medical aspects of the Insurance Bill. The many merits of this measure are clearly apparent to the author, whose experience as an administrative officer enables him to see also all the weak points of the scheme in its actual operation. The case for the profession is here presented not in any narrow spirit, but with a knowledge of the conditions of present-day medicine and of present-day general practice, which enables the author to appreciate clearly the difficulties under which the profession labours and the way in which the Bill may increase those difficulties. We do not think any layman who reads these chapters will accuse the profession of any sordid desire for gain; and we feel sure that he will end up with a much deeper insight into the complexities of national insurance and into the folly and futility of trying to deal with them offhand without the most mature consideration and the fullest discussion.

Diseases of the Nose and Throat. By St. Clair Thomson, M.D., F.R.C.P., F.R.C.S. (London: Cassell and Co., Ltd., 25s.)

Dr. Thomson's new manual of diseases of the nose and throat is a triumph of condensation, and one of the best text-books on the subject which we have read. This is high praise when we bear in mind that there are already very excellent rivals, especially in German, on the market, but it is not exaggerated, since the book is so well illustrated and so carefully written that it can truly be called a model text-book. It is obviously not a specialist's manual; the references, though copious, are not exhaustive, and the author is at some pains to deal very thoroughly with the more elementary aspects of the subject. For this reason, all the more, it is a very desirable book for the student and general practitioner, and admirable as a compendium. A little more might with advantage have been written concerning the after-treatment, especially in such common conditions as intubation and tracheotomy. With this exception there is nothing to cavil at; the book is a complete summary, of real help to the practitioner who consults it.

Naturally the subjects, then, which will claim the first consideration are those which refer to common everyday conditions. Perhaps the most important of these, from the practitioner's point of view, is adenoids. Dr. Thomson considers the prognosis after operation in these cases in a short paragraph which should be carefully weighed by every practitioner. "The results are generally so excellent that there need be no hesitation in recommending it (the operation for removal) whenever indicated." The reader finds the indications mentioned on the same page. Briefly, they are: mouth breathing in the day; frequent mouth breathing at night; night terrors; chronic concomitant conditions of the upper air passages, enlarged glands; affections of the ear; spasmodic cough; threatened malformation of the chest and hard palate; and certain neuroses which "cannot be explained and relieved by reference to other factors." The first two indications seem to us insufficient in themselves to demand the operation. Mouth breathing is in the majority of cases a matter of habit, and may be cured by proper exercises. Dr. Thomson prefers to operate under an anæsthetic, but explains that there is a certain amount of risk in doing so. The chapter on tonsils is equally full, and contains many valuable hints. The indications herein given for removal are excellent, and we note with satisfaction that the author lays stress on a point so often overlooked by practitioners —namely, that it is the septic state of the tonsils, rather than their size, which often determines the question of removal. With regard to enucleation, it is worth while pointing out that serious contraction by scar tissue has sometimes followed the operation.

It is obviously impossible to deal with the various chapters of the book, or even to attempt to deal with them, within the limits of a brief review. All that can be attempted here is to point out that the volume is in every respect suitable to be added to the practitioner's library.

